# Theoretical and Numerical Investigation of Normalized Sensitivity in MEMS Capacitive Pressure Sensors Based on Dimensionless Large-Deflection Governing Equations

**DOI:** 10.3390/mi17080941

**Published:** 2026-08-07

**Authors:** Xiaoda Cao, Haiwang Li, Shizhe Song, Tiantong Xu, Yanxin Zhai

**Affiliations:** 1 School of Energy and Power Engineering, Beihang University, Beijing 100191, China; buaacxd@buaa.edu.cn; 2 The Research Institute of Aero-Engine, Beihang University, Beijing 100191, China; 09620@buaa.edu.cn (H.L.); xutiantong@buaa.edu.cn (T.X.); 3 Taihang Laboratory, Chengdu 610000, China; song_shizhe@163.com

**Keywords:** MEMS, capacitive pressure sensors, normalized sensitivity, large-deflection governing equations

## Abstract

MEMS capacitive pressure sensors are one of the major classes of pressure sensors. However, in the non-contact operating mode, their sensitivity often varies nonlinearly with the applied pressure, which increases the difficulty and workload of sensitivity evaluation at multiple pressure points during the design stage. In this study, a normalized sensitivity analysis framework for MEMS capacitive pressure sensors is established based on the large-deflection governing equations. Within this framework, the nonlinear relationship between normalized sensitivity and normalized pressure is characterized by five dimensionless parameters. The results show that, when these dimensionless parameters are identical, sensors with different combinations of structural and material parameters collapse onto nearly identical normalized sensitivity-pressure characteristic curves, with an average relative deviation not exceeding 0.05% in the verification cases. In addition, the effects of each dimensionless parameter on the nonlinear behavior of the characteristic curves are further analyzed. The proposed framework provides a unified and systematic analytical basis for sensitivity comparison, performance evaluation, and structural optimization of MEMS capacitive pressure sensors.

## 1. Introduction

MEMS pressure sensors are widely used miniaturized devices for pressure measurement. Common implementations include piezoresistive, capacitive, fiber-optic, and resonant types [1,2,3]. Among these, capacitive pressure sensors have been extensively studied and deployed in industrial control, automotive electronics, and aerospace applications [4,5,6]. This is primarily attributable to their low power consumption, high sensitivity, good temperature stability, and suitability for batch fabrication [7,8,9]. Their basic operating principle is as follows. A pressure-sensing diaphragm bends under an applied pressure load. The resulting deflection changes the electrode gap and therefore the capacitance. In this way, a pressure signal is converted into a capacitance signal. Sensitivity is one of the most important performance metrics for this class of sensors. It should be quantified during the design stage to guide the selection of structural dimensions and material parameters.

Existing studies typically obtained the sensitivity through analytical modeling and numerical computation. In analytical modeling, the diaphragm deflection under a uniformly distributed load is first solved using either the small-deflection equation or the large-deflection equations. The capacitance is then obtained by area integration over the deformed electrode gap [10,11,12,13]. Numerical computation commonly employs the finite element method to first solve the diaphragm deflection field and then extract the capacitance via area integration, or to directly obtain the capacitance response through multiphysics coupling [14,15,16,17]. Sensitivity is typically evaluated at multiple pressure points across the full-scale range, either by finite-difference approximation of the local capacitance response or by differentiating the capacitance-pressure relationship. However, comparing full-range sensitivity characteristics across different design-parameter combinations generally requires repeated point-by-point capacitance and sensitivity evaluations, thereby increasing the computational workload during iterative sensor design and parameter optimization.

To further clarify the influence of individual design parameters on the sensitivity of capacitive pressure sensors, prior studies have progressively introduced normalized analysis for capacitive pressure sensors. Rochus et al. derived a rapid sensitivity analysis model for circular-diaphragm sensors under the small-deflection assumption [18,19]. They used the sensitivity as the pressure difference approaches zero as a characteristic point (initial sensitivity). Building on this work, Jindal et al. normalized the operating pressure and sensitivity using the contact pressure and initial sensitivity as reference quantities [20]. They then proposed a sensitivity characteristic curve for circular-diaphragm capacitive pressure sensors. Xu et al. further considered the influence of the insulating layer on the nonlinear characteristics of circular-diaphragm capacitive pressure sensors [21]. These studies primarily focus on circular-diaphragm structures under the small-deflection assumption. However, systematic analysis and unified characterization remain lacking for the effects of large-deflection operation and structural factors such as diaphragm geometry on normalized sensitivity.

In this study, a normalized sensitivity analysis framework for capacitive pressure sensors is developed based on the governing equations for large deflection. The large-deflection governing equations are first nondimensionalized. The operating pressure and sensitivity are then normalized. From this formulation, the dimensionless parameters that govern the normalized sensitivity-pressure characteristic curves are identified, and their influences on the nonlinear behavior of the characteristic curves are analyzed. This framework enables rapid evaluation and comparison of sensitivity characteristics across different parameter combinations, thereby providing an efficient analytical basis for sensor structural design and parameter optimization.

## 2. Materials and Methods

### 2.1. Basic Sensor Model

**Figure 1:**
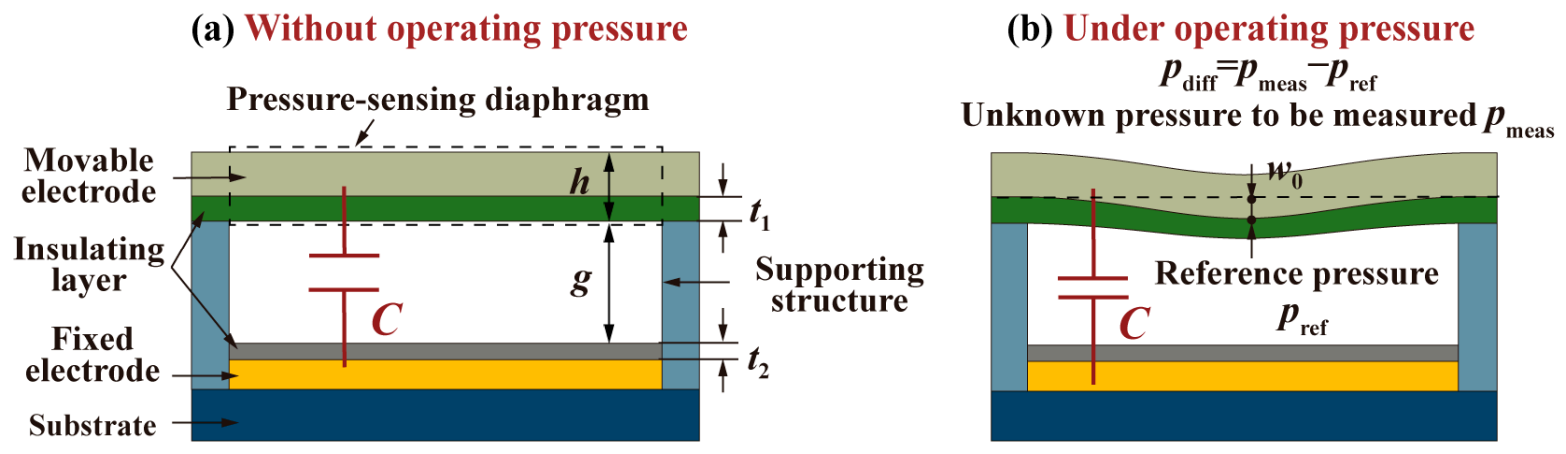
Schematic diagram of the capacitive pressure sensor structure. (**a**) Without operating pressure. (**b**) Under operating pressure.

A typical two-dimensional structure of a capacitive pressure sensor is shown in Figure 1. As illustrated in Figure 1a, the sensor consists of a pressure-sensitive diaphragm, an electrode gap, a fixed electrode, an insulating layer, a supporting structure, and a substrate. The diaphragm has a thickness *h*, and the movable electrode is part of the diaphragm. The electrode gap has a height *g*. The insulating layer is typically located either on the bottom surface of the movable electrode (thickness *t*_1_, permittivity *ε*_ins1_) or on the top surface of the fixed electrode (thickness *t*_2_, permittivity *ε*_ins2_) [17,22]. The sensing capacitance is formed between the movable and fixed electrodes. As shown in Figure 1b, the measured pressure on the upper surface of the diaphragm is *p*_meas_ and the reference pressure on the lower surface is *p*_ref_. The pressure difference across the diaphragm *p*_diff_ is defined as the operating pressure of the sensor.

(1) $$p_{\mathrm{diff}}=p_{\mathrm{meas}}-p_{\mathrm{ref}}$$

When subjected to *p*_diff_, the diaphragm deflects, and the resulting central deflection is denoted by *w*_0_.

**Figure 2:**
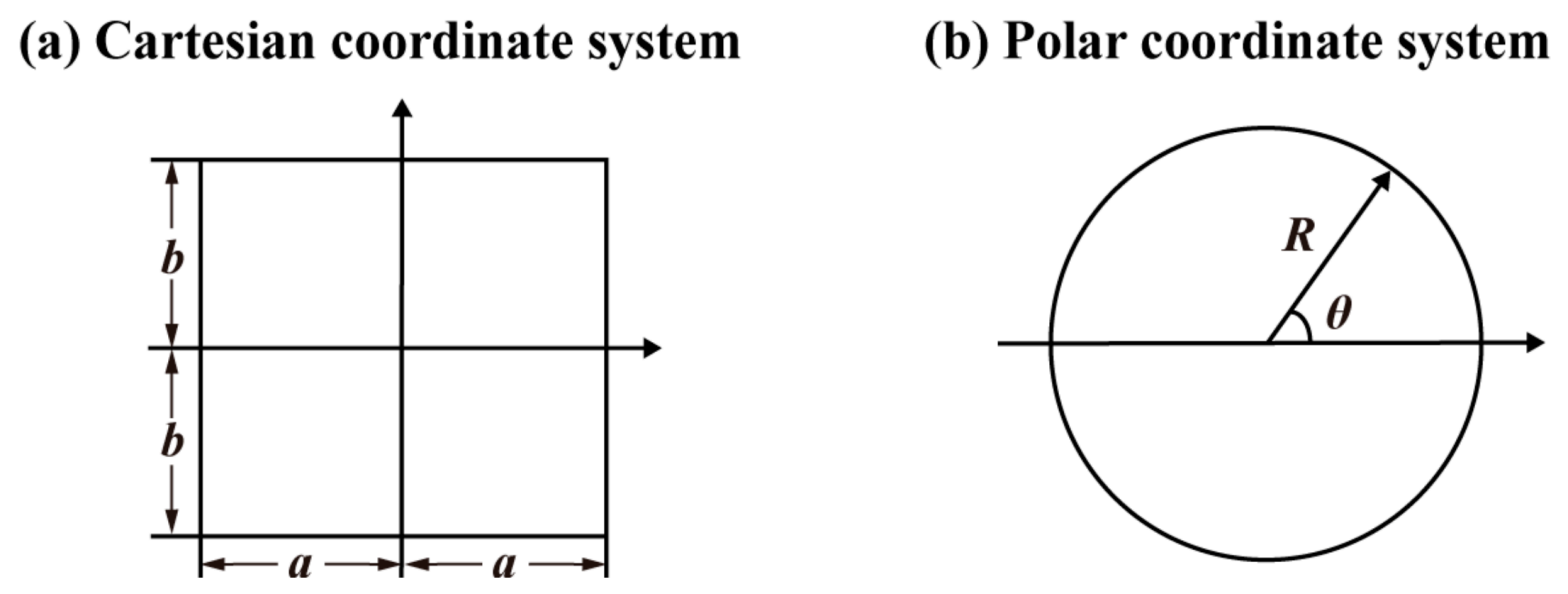
Coordinate systems of the pressure-sensing diaphragm. (**a**) Cartesian coordinate system. (**b**) Polar coordinate system.

The coordinate systems used to describe the diaphragm deflection field are defined in Figure 2. Common diaphragm geometries include square, rectangular, and circular plates [23,24,25]. Square and rectangular diaphragms are described in a Cartesian coordinate system [Figure 2a], with *x* and *y* as the coordinate variables. Circular diaphragms are formulated in a polar coordinate system [Figure 2b], with *r* and *θ* as the coordinate variables. In all cases, the coordinate origin is located at the geometric center of the diaphragm.

For a rectangular diaphragm, the short side and long side are denoted by *a* and *b*, respectively. The aspect ratio is defined as *AR* = *b*/*a* with *AR* ≥ 1. The square diaphragm is a special case with *AR* = 1. Accordingly, the diaphragm deflection can be expressed as *w*(*x*, *y*).

Under the local parallel-plate approximation and the quasi-static electric-field assumption, with fringe-field effects neglected, the output capacitance *C* of a rectangular-diaphragm sensor is given as follows.

(2) $$C=\int_{-a}^{a}\int_{-b}^{b}\frac{\varepsilon_{0}\varepsilon_{\mathrm{ins}}\varepsilon_{a}}{\varepsilon_{a}t_{\mathrm{ins}}+\varepsilon_{\mathrm{ins}}\left(g-w(x,y)\right)}dxdy$$

Here, *ε*_ins_ is defined as the effective relative permittivity of the dielectric layer, and *t* is the corresponding effective thickness. They are derived by treating the dielectric layers on the fixed-electrode surface and the bottom surface of the movable electrode as a series stack along the thickness direction. The resulting expressions are provided below.

(3) $$\varepsilon_{\mathrm{ins}}=\varepsilon_{\mathrm{ins}1}\varepsilon_{\mathrm{ins}2}$$

(4) $$t=\varepsilon_{\mathrm{ins}1}t_{2}+\varepsilon_{\mathrm{ins}2}t_{1}$$

For a circular-diaphragm sensor with diaphragm radius *R*, the output capacitance in the polar coordinate system is given as follows.

(5) $$C=\int_{0}^{2\pi }\int_{0}^{R}\frac{\varepsilon_{0}\varepsilon_{\mathrm{ins}}\varepsilon_{a}r}{\varepsilon_{a}t+\varepsilon_{\mathrm{ins}}\left(g-w(r)\right)}drd\theta$$

In principle, the sensitivity *S*(*p*_diff_) can be obtained by differentiating Equations (2) and (5) with respect to *p*_diff_. During this process, the deflection function *w* appearing in Equations (2) and (5) must be determined in advance. For the theoretical solution of the diaphragm deflection *w*, the pressure-sensing diaphragm is typically idealized as a thin plate. The plate is assumed to be linear elastic and isotropic, with uniform thickness and no initial warpage. The plate is modeled with fully clamped boundaries along all edges and is subjected to a transverse load *p*_diff_. Furthermore, the plate deformation can be described by the large-deflection governing equations.

(6) $$D\nabla^{4}w=h(\frac{\partial^{2}\phi }{\partial x^{2}}\frac{\partial^{2}w}{\partial y^{2}}+\frac{\partial^{2}\phi }{\partial y^{2}}\frac{\partial^{2}w}{\partial x^{2}}-2\frac{\partial^{2}\phi }{\partial x\partial y}\frac{\partial^{2}w}{\partial x\partial y})+p_{\mathrm{diff}}$$

(7) $$\nabla^{4}\phi =E\left[{\left(\frac{\partial^{2}w}{\partial x\partial y}\right)}^{2}-\frac{\partial^{2}w}{\partial x^{2}}\frac{\partial^{2}w}{\partial y^{2}}\right]$$

Here, *ϕ* denotes the stress function. *E* is the Young’s modulus of the diaphragm, and *ν* is its Poisson’s ratio. *D* represents the flexural rigidity, defined as follows.

(8) $$D=\frac{Eh^{3}}{12(1-\nu^{2})}$$

Accounting for the effect of an initial uniform biaxial residual stress *σ*_0_ in the thin plate, Equation (6) is modified to Equation (9).

(9) $$D\nabla^{4}w=h(\frac{\partial^{2}\phi }{\partial x^{2}}\frac{\partial^{2}w}{\partial y^{2}}+\frac{\partial^{2}\phi }{\partial y^{2}}\frac{\partial^{2}w}{\partial x^{2}}-2\frac{\partial^{2}\phi }{\partial x\partial y}\frac{\partial^{2}w}{\partial x\partial y})+h\sigma_{0}\nabla^{2}w+p_{\mathrm{diff}}$$

Owing to the complexity of the large-deflection governing equations, an explicit analytical relationship between the deflection *w* and *p*_diff_ is difficult to derive. For each specified *p*_diff_, the corresponding deflection field *w* is typically obtained by solving the governing equations separately. As a result, a closed-form analytical expression for the sensitivity *S*(*p*_diff_) of the capacitive pressure sensor is generally unavailable, except for circular diaphragms under the small-deflection assumption. Therefore, the sensitivity at a given operating pressure *p*_diff_, denoted as *S*(*p*_diff_), is evaluated using the following finite-difference approximation.

(10) $$S(p_{\mathrm{diff}})=\frac{C(p_{\mathrm{diff}}+\Delta p)-C(p_{\mathrm{diff}})}{\Delta p}$$

Here, Δ*p* represents a prescribed small pressure increment. Consequently, evaluating the sensitivity at multiple operating points within the sensor’s operating range generally requires repeated computations, leading to a considerable computational burden and limiting the efficiency of iterative sensor design.

In view of the above considerations, a normalized sensitivity analysis is proposed for capacitive pressure sensors, with the aim of characterizing their sensitivity behavior under different combinations of design parameters.

### 2.2. Nondimensionalization of the Large-Deflection Governing Equations

As indicated by the above analysis, *w* acts as an intermediate variable linking *S*(*p*_diff_) and *p*_diff_. Accordingly, the relationship between *w* and *p*_diff_ is first examined based on the nondimensionalized large-deflection governing equations, providing a basis for the subsequent establishment of the relationship between *p*_diff_ and *S*(*p*_diff_). The deflection solution *w* of the large-deflection governing equations can be represented as follows.

(11) $$w(x,y)=w_{0}W(x,y)$$

Here, *w*_0_ is the maximum deflection and *W*(*x*,*y*) denotes the deflection shape function, which takes values in the range [0, 1]. For a circular diaphragm, its deflection shape function can be expressed as *W*(*r*). Both *W*(*x*,*y*) and *W*(*r*) can be approximated by a polynomial expression, as shown in Equation (12).

(12) $$W(x,y)={\left(1-\frac{x^{2}}{a^{2}}\right)}^{2}{\left(1-\frac{y^{2}}{b^{2}}\right)}^{2}(c_{0}+c_{1}\frac{x^{2}}{a^{2}}+c_{2}\frac{y^{2}}{b^{2}}+\ldots )$$

We choose the characteristic scales *L*_ix_ = *a*, *L*_iy_ = *b*, *w*_i_ = *h*, *p*_i_ = *Eh*^4^/*a*^4^, *σ*_i_ = *Eh*^2^/*a*^2^ and *ϕ*_i_ *= Eh*^2^ for the coordinates, deflection, operating pressure, residual stress, and the stress function, respectively. The characteristic scales are selected based on the order-of-magnitude balance arising from the nondimensionalization of the large-deflection governing equations, while also ensuring dimensional consistency with the corresponding physical quantities. The corresponding dimensionless variables are defined as follows.

(13) $$x^{*}=\frac{x}{L_{\mathrm{ix}}},y^{*}=\frac{y}{L_{\mathrm{iy}}},w_{0}^{*}=\frac{w_{0}}{w_{i}},p^{*}=\frac{p_{\mathrm{diff}}}{p_{i}},\sigma^{*}=\frac{\sigma_{0}}{\sigma_{i}},\phi^{*}=\frac{\phi }{\phi_{i}}$$

By substituting Equation (13) into Equations (7) and (9), the corresponding dimensionless forms are obtained.

(14) $$\begin{array}{ll} \frac{w_{0}^{*}}{12(1-v^{2})}\nabla^{*4}W^{*}(x^{*},y^{*}) & =\frac{w_{0}^{*}}{AR^{2}}(\frac{\partial^{2}\phi^{*}}{\partial x^{*2}}\frac{\partial^{2}W^{*}(x^{*},y^{*})}{\partial y^{*2}}+\frac{\partial^{2}\phi^{*}}{\partial y^{*2}}\frac{\partial^{2}W^{*}(x^{*},y^{*})}{\partial x^{*2}}\\ & -2\frac{\partial^{2}\phi^{*}}{\partial x^{*}\partial y^{*}}\frac{\partial^{2}W^{*}(x^{*},y^{*})}{\partial x^{*}\partial y^{*}})+\sigma^{*}w_{0}^{*}\nabla^{*2}W^{*}(x^{*},y^{*})+p^{*} \end{array}$$

(15) $$\nabla^{*4}\phi^{*}=\frac{w_{0}^{*2}}{AR^{2}}\left[{\left(\frac{\partial^{2}W^{*}(x^{*},y^{*})}{\partial x^{*}\partial y^{*}}\right)}^{2}-\frac{\partial^{2}W^{*}(x^{*},y^{*})}{\partial x^{*2}}\frac{\partial^{2}W^{*}(x^{*},y^{*})}{\partial y^{*2}}\right]$$

The relationship between the dimensionless deflection shape function W^*^(x^*^, y^*^) and the deflection shape function *W*(*x*,*y*) is given as follows.

(16) $$W^{*}(\frac{x}{L_{\mathrm{ix}}},\frac{y}{L_{\mathrm{iy}}})=W(x,y)$$

Within the range of p^*^ where instability (e.g., buckling) does not occur, each prescribed value of p^*^ is generally assumed to correspond to a unique stable equilibrium solution of the form *w*_0_^*^W^*^(x^*^, y^*^). For rectangular diaphragms, this mapping is determined by the prescribed values of *ν*, *AR*, and σ^*^. For circular diaphragms, however, the aspect ratio *AR* is not defined, and the mapping depends only on *ν* and σ^*^. Furthermore, the dimensionless deflection shape function W^*^(x^*^, y^*^) is normalized such that its maximum value equals unity. Consequently, for each prescribed value of p^*^, there exists a unique corresponding value of *w*_0_^*^. Moreover, based on fundamental physical considerations, *w*_0_^*^ is expected to increase monotonically with increasing p^*^. The relationship between *w*_0_^*^ and p^*^ is described by Equation (17).

(17) $$p^{*}=f(w_{0}^{*})$$

In addition, within the small-deflection regime, the relationship between p^*^ and *w*_0_^*^ exhibits a simple linear dependence.

Overall, based on the large-deflection governing equations, this section identifies the dimensionless parameters governing the p^*^−*w*_0_^*^ relationship and the dependence of the normalized deflection shape function W^*^(x^*^, y^*^) on p^*^. It should be noted that this section focuses solely on the mechanical response of the diaphragm itself, without yet incorporating factors related to the sensor’s pressure range or capacitive response.

### 2.3. Normalization Procedure

To normalize the relationship between *p*_diff_ and *S*(*p*_diff_), the contact pressure *p*_c_ and the initial sensitivity *S*_0_ are selected as the baseline values. For a capacitive pressure sensor operating in the non-contact mode, *p*_c_ defines the theoretical upper limit of the operating range, corresponding to the pressure at which the diaphragm first comes into contact with the gap surface, *w*_0_ = *g*. According to Equation (17), the normalized contact pressure *p*_c_^*^ can be expressed as follows.

(18) $$p_{c}^{*}=f(\frac{g}{h})=f(\lambda )$$

Here, *λ* denotes the gap-to-thickness ratio, defined by *λ* = *g*/*h*. According to the analysis of the p^*^−*w*_0_^*^ relationship in Section 2.2 and Equation (18), the value of *p*_c_^*^ is uniquely determined for prescribed values of *ν*, *AR*, σ^*^, and *λ*. Moreover, *p*_c_ is given as follows.

(19) $$p_{c}=\frac{Eh^{4}}{a^{4}}f(\frac{g}{h})$$

With *p*_c_ as the normalization base, the normalized operating pressure *p*_n_ is defined as follows.

(20) $$p_{n}=\frac{p_{\mathrm{diff}}}{p_{c}}=\frac{p^{*}}{p_{c}^{*}}$$

In light of the above analysis, Equation (2) can be reformulated as follows.

(21) $$C(p_{n})=\frac{C_{0}}{4}\int_{-1}^{1}\int_{-1}^{1}\frac{1}{1-\gamma w_{n}(p_{n})W^{*}(x^{*},y^{*})}dx^{*}dy^{*}$$

Here, *γ* denotes the equivalent gap coefficient, which is defined as follows.

(22) $$\gamma =\frac{\varepsilon_{\mathrm{ins}}g}{\varepsilon_{a}t+\varepsilon_{\mathrm{ins}}g}$$

And *w*_n_ is defined as the normalized deflection, and its functional relationship with *p*_n_ can be expressed as follows.

(23) $$w_{n}(p_{n})=\frac{w_{0}}{g}=\frac{{w_{0}}^{*}}{\lambda }=\frac{f^{-1}(p_{n}f(\lambda ))}{\lambda }$$

In the small-deflection regime, the normalized operating pressure *p*_n_ is directly equal to the normalized deflection *w*_n_.

Overall, for sensors with identical values of *ν*, *AR*, σ^*^, and *λ*, at a given normalized operating pressure *p*_n_, the corresponding dimensionless pressure p^*^ = *p*_n_*p*_c_^*^ is therefore identical, resulting in the same dimensionless equilibrium deflection field. Specifically, the normalized maximum deflection at *p*_n_ satisfies *w*_0_^*^(*p*_n_) = *λw*_n_(*p*_n_), and the corresponding deflection field can be expressed as *w*_0_^*^(*p*_n_)W^*^(x^*^, y^*^) = *λw*_n_(*p*_n_)W^*^(x^*^, y^*^).

In addition, *C*_0_ denotes the actual initial capacitance of the sensor at zero operating pressure, rather than an arbitrary capacitance scale factor. The expression for *C*_0_ of the rectangular diaphragm sensor is given as follows.

(24) $$C_{0}=\frac{4\varepsilon_{0}\varepsilon_{a}\gamma ab}{g}$$

For a circular diaphragm sensor, *C*_0_ can be expressed as follows.

(25) $$C_{0}=\frac{\pi \varepsilon_{0}\varepsilon_{a}\gamma R^{2}}{g}$$

Furthermore, the normalized capacitance *C*_n_(*p*_n_) can be expressed as follows.

(26) $$C_{n}(p_{n})=\frac{C(p_{n})}{C_{0}}$$

In fact, *C*_n_(*p*_n_) is given by one quarter of the integral term in Equation (21). Therefore, for sensors with different physical parameters, the mapping between *C*_n_ and *p*_n_ is identical as long as *ν*, *AR*, σ^*^, *λ*, and *γ* are fixed.

To normalize the sensitivity, this study selects the sensitivity at *p*_diff_ ≈ 0 as the baseline sensitivity *S*_0_, which is expressed as follows.

(27) $$S_{0}=\frac{C(\Delta p)-C(0)}{\Delta p}$$

For a small Δ*p*, the normalized pressure increment Δ*p*_n_ = Δ*p*/*p*_c_ is small, and thus the resulting *w*_n_(Δ*p*_n_) is also small. Therefore, *C*(Δ*p*) can be expanded in a Taylor series as follows.

(28) $$C_{n}\left(\Delta p_{n}\right)=\frac{1}{4}\int_{-1}^{1}\int_{-1}^{1}\left(1+\gamma w_{n}(\Delta p_{n})W^{*}(x^{*},y^{*})\right)dx^{*}dy^{*}$$

By substituting Equation (28) into Equation (27), the expression of the baseline sensitivity *S*_0_ can be obtained as follows.

(29) $$S_{0}=\frac{\gamma C_{0}\int_{-1}^{1}\int_{-1}^{1}w_{n}(\Delta p_{n})W^{*}(x^{*},y^{*})dx^{*}dy^{*}}{4p_{c}\Delta p_{n}}$$

Furthermore, the normalized sensitivity of the sensor, *S*_n_(*p*_n_) can be expressed as follows.

(30) $$S_{n}=\frac{S\left(p_{\mathrm{diff}}\right)}{S_{0}}=\frac{4[C_{n}\left(p_{n}+\Delta p_{n}\right)-C_{n}\left(p_{n}\right)]}{\gamma \int_{-1}^{1}\int_{-1}^{1}w_{n}(\Delta p_{n})W^{*}(x^{*},y^{*})dx^{*}dy^{*}}$$

It can be concluded that when Δ*p*_n_, *ν*, *AR*, σ^*^, *λ*, and *γ* are fixed, the normalized sensitivity *S*_n_ given by the above equation depends only on the normalized operating pressure *p*_n_. In general, Δ*p*_n_ can be prescribed as a fixed fraction of the full-scale range, such as 0.001. Therefore, during the design process, the nonlinear sensitivity characteristics over the full operating range in the non-contact mode can be regarded as being determined by *ν*, *AR*, σ^*^, *λ*, and *γ*.

This means that, to compare the full-scale sensitivity among different sensors, it is sufficient to obtain the baseline sensitivity *S*_0_ (i.e., the sensitivity evaluated at the single pressure point *p*_diff_ ≈ 0) together with the aforementioned dimensionless parameters. Consequently, it is unnecessary to compute the sensitivity point by point over multiple pressure levels for sensors with different physical parameters.

Furthermore, under small-deflection conditions, Equation (29) reduces to Equation (31).

(31) $$S_{0}=\frac{\gamma C_{0}a^{4}}{4\lambda KEh^{4}}\int_{-1}^{1}\int_{-1}^{1}W^{*}(x^{*},y^{*})dx^{*}dy^{*}$$

Here, *K* is the constant coefficient of the linear relationship between p^*^ and *w*_0_^*^ in the small-deflection regime, indicating that p^*^ is *K* times *w*_0_^*^. Accordingly, Equation (30) can be rewritten as follows.

(32) $$S_{n}\left(p_{n}\right)=\frac{\int_{-1}^{1}\int_{-1}^{1}\frac{W^{*}(x^{*},y^{*})}{{\left[1-\gamma p_{n}W^{*}(x^{*},y^{*})\right]}^{2}}dx^{*}dy^{*}}{\int_{-1}^{1}\int_{-1}^{1}W^{*}(x^{*},y^{*})dx^{*}dy^{*}}$$

Since *λ* is absent from the *S*_n_−*p*_n_ relationship, sensors with identical values of *ν*, *AR*, σ^*^, and *γ* exhibit the same normalized sensitivity characteristics under small-deflection conditions. In general, the small-deflection regime corresponds to *w*_0,max_/*h* < 0.2.

### 2.4. Indirect Computation Method for Normalized Sensitivity−Pressure Characteristic Curves

Based on the above derivation and conclusions regarding the *S*_n_−*p*_n_ relationship of capacitive pressure sensors, this study proposes an indirect approach for computing the *S*_n_−*p*_n_ correspondence. The core idea follows directly from the preceding analysis: when the key dimensionless parameters are identical, the *S*_n_−*p*_n_ relationship is invariant among different capacitive pressure sensors. Therefore, a general normalized sensitivity curve can be obtained from a limited number of representative simulation cases.

First, a set of physical geometric and material parameters (*h*, *g*, *a*, *b*, *σ*_0_, *t*, *ε*_ins_, *ν*, *E*) is selected such that the prescribed dimensionless parameters (*ν*, *AR*, σ^*^, *λ*, and *γ*) are satisfied, and a corresponding simulation model is constructed. Next, the contact pressure *p*_c_ is determined numerically from the FEA model as the pressure at which the central deflection reaches the capacitive gap, *w*_0_ = *g*. The normalized operating pressure *p*_n_ is then discretized over the interval [0, 1) into a specified number of sampling points. For each *p*_n_, the two corresponding operating pressures are computed as *p*_n_*p*_c_ and (*p*_n_ + Δ*p*_n_)*p*_c_, where Δ*p*_n_ is taken as a fixed increment. In all numerical calculations throughout this study, the prescribed normalized pressure increment Δ*p*_n_ is uniformly set to 0.001, corresponding to 0.1% of the contact-pressure range. These two pressures are applied as surface loads on the diaphragm in the simulation model, yielding the capacitances *C*(*p*_n_*p*_c_) and *C*((*p*_n_ + Δ*p*_n_)*p*_c_). Finally, the normalized sensitivity *S*_n_(*p*_n_) at each sampling point *p*_n_ is evaluated using the following expression.

(33) $$S_{n}\left(p_{n}\right)=\frac{C((p_{n}+\Delta p_{n})p_{c})-C(p_{n}p_{c})}{C(\Delta p_{n}p_{c})-C(0)}$$

### 2.5. Simulation Setup

**Figure 3:**
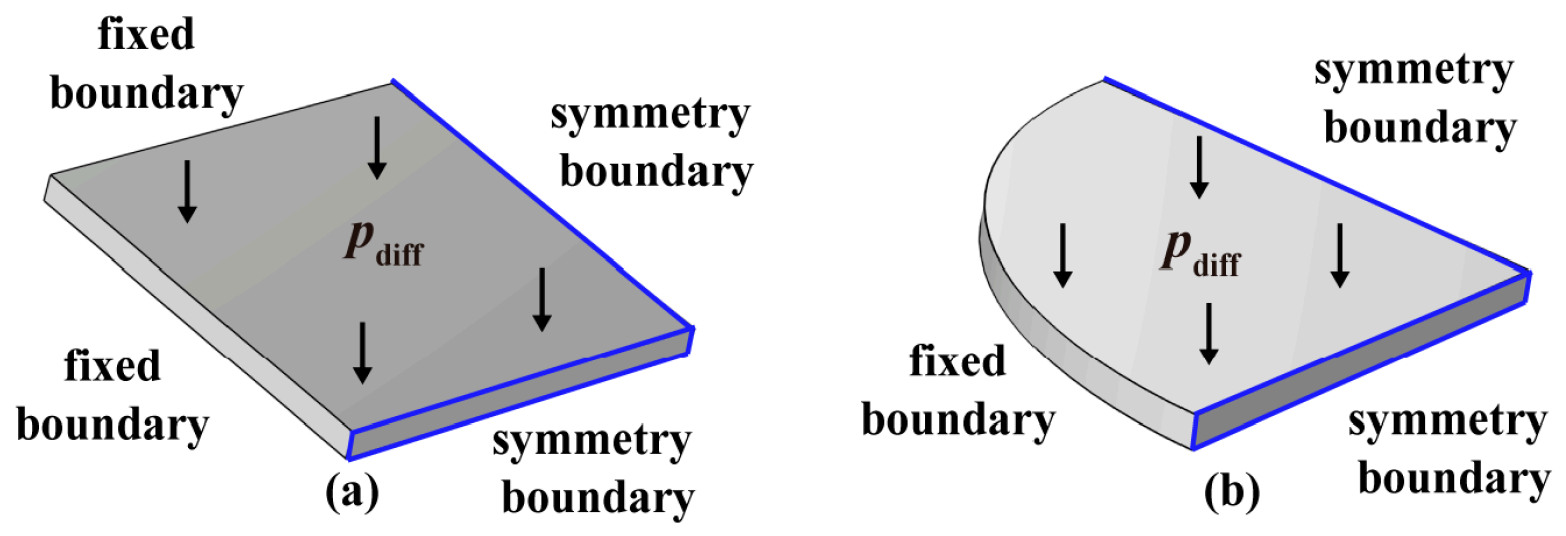
Diaphragm deflection simulation model. (**a**) rectangular diaphragm. (**b**) circular diaphragm.

This study performs simulations only for the pressure-sensitive diaphragm itself, while omitting nonessential components such as the supporting structure. The simulated configurations include clamped rectangular and circular diaphragms. Fixed boundary conditions are imposed on the diaphragm sidewalls, and a spatially uniform pressure *p*_diff_ is applied normal to the top surface of the diaphragm. A quarter of the diaphragm is selected for analysis, and symmetry boundary conditions are imposed on the two cross-sectional planes of the quarter model as indicated by the blue-outlined regions in Figure 3a,b.

The numerical simulations are performed using a stationary solid-mechanics finite-element formulation, with geometric nonlinearity included to account for the large-deflection behavior of the diaphragm. A free quadrilateral mesh is first generated on the diaphragm surface and then swept through the diaphragm thickness to produce hexahedral elements. Mesh independence is evaluated using the contact pressure *p*_c_, defined as the pressure at which the central diaphragm deflection reaches the capacitive gap *w*_0_ = g, as the convergence criterion. Taking Sensor 1 in Section 3.1 as an example, five meshes containing 4800, 9600, 18,700, 38,236, and 78,196 elements are examined. With the result obtained using 78,196 elements taken as the reference, the relative deviations in *p*_c_ obtained using all the coarser meshes are less than 0.5%, indicating that the predicted contact pressure is insensitive to further mesh refinement. Therefore, the mesh containing approximately 4800 elements is adopted to balance computational accuracy and efficiency. The same mesh-independence assessment is performed for all other simulation cases, with the relative deviations in *p*_c_ consistently below 0.5%.

To obtain the sensor capacitance, the capacitance contributions associated with the surface area elements on the lower surface of the quarter-diaphragm domain are integrated according to Equation (2). Owing to structural symmetry, the integral result is multiplied by four to yield the total capacitance.

## 3. Results and Discussion

First, the normalized sensitivity characteristics of sensors with different dimensional geometric and material parameters but identical governing dimensionless parameters are compared. The agreement among the resulting *S*_n_−*p*_n_ curves is used to verify the preceding conclusion regarding the consistency of normalized sensitivity characteristics within the proposed dimensionless framework. Subsequently, the influence of each dimensionless parameter on the normalized sensitivity characteristics is analyzed.

### 3.1. Verification of Similarity in Normalized Sensitivity Characteristics

For the circular-diaphragm sensors, Sensors 1 and 2 with *γ* = 0.9647, *λ* = 0.7, *ν* = 0.28, and σ^*^ = −0.2 are selected for simulation. For the rectangular-diaphragm sensors, Sensors 3 and 4 with *γ* = 0.9474, *AR* = 2, *λ* = 1.8, *ν* = 0.28, and σ^*^ = −0.5 are selected for simulation. The specific geometric and material parameters are listed in Table 1.

**Figure 4:**
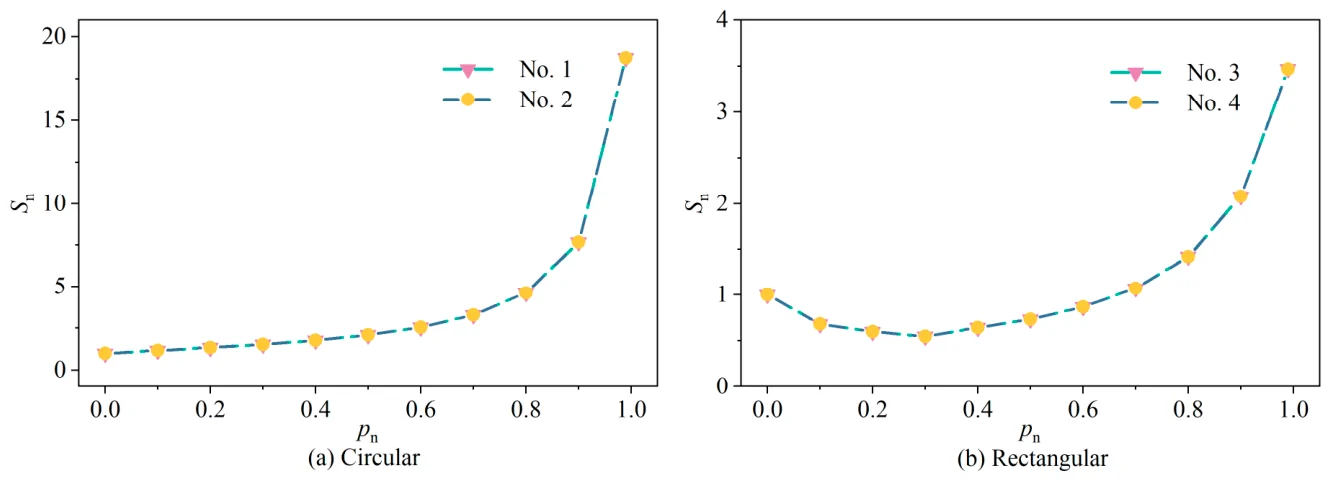
Verification of the similarity of the sensor *S*_n_−*p*_n_ curves. (**a**) circular diaphragm. (**b**) rectangular diaphragm (*AR* = 2).

The *S*_n_−*p*_n_ curves for the two sensor groups are shown in Figure 4. The normalized sensitivity *S*_n_ is evaluated at 11 representative pressure points covering the entire investigated range, namely *p*_n_ = 0, 0.1, 0.2, …, 0.9, and 0.99, to characterize the overall nonlinear evolution of the *S*_n_−*p*_n_ relationship. At each pressure point, the absolute relative deviation is calculated using Sensor 2 as the reference for the circular-diaphragm case and Sensor 4 as the reference for the rectangular-diaphragm case. The average relative deviation is then obtained by averaging the absolute relative deviations at all 11 pressure points.

For the circular-diaphragm sensors shown in Figure 4a, the average relative deviation of Sensor 1 from Sensor 2 is only 0.01%. Further comparison of the calculated data shows that the maximum relative deviation is 0.04%, occurring at *p*_n_ = 0.99. For the rectangular-diaphragm sensors shown in Figure 4b, the average relative deviation of Sensor 3 from Sensor 4 is 0.05%, while the maximum relative deviation is 0.13% at *p*_n_ = 0.99. Although the deviations increase slightly near the upper end of the pressure range, they remain very small throughout the investigated range. These results verify the theoretical similarity of the normalized sensitivity-pressure relationships and further confirm the applicability of the proposed indirect computation method.

### 3.2. Effects of Key Dimensionless Parameters

In this section, the influences of the five dimensionless parameters, *λ*, *AR*, *ν*, σ^*^, and *γ*, on the normalized sensitivity characteristics are systematically examined. All results are obtained using the indirect computation method proposed in Section 2.4.

The range of *λ* is determined based on the typical dimensions of silicon capacitive pressure sensors. The diaphragm thickness *h* typically ranges from several micrometers to several tens of micrometers, whereas the gap *g* is generally on the order of a few micrometers. In addition, thinner diaphragms are generally preferred to achieve higher sensitivity. Based on these typical dimensions and design considerations, *λ* is chosen to vary from 0 to 2 in this study.

For the aspect ratio *AR*, rectangular diaphragms with *AR* = 1, 2, and 3, together with a circular diaphragm, are considered to represent a broad range of commonly used diaphragm geometries.

For the Poisson’s ratio *ν*, *ν* = 0.28 is first adopted to represent silicon, the primary structural material of the diaphragm in silicon capacitive pressure sensors. Considering the possible presence of insulating layers with lower Poisson’s ratios, such as silicon dioxide and silicon nitride, *ν* = 0.22 is further introduced as a comparison case.

For the dimensionless residual stress σ^*^, the stress-free condition σ^*^ = 0 is first adopted as the baseline case. In addition, σ^*^ = 0.5, 0.25, −0.25 and σ^*^ = −0.50 are considered as comparison cases to evaluate the influence of residual stress. The present analysis assumes that the diaphragm remains initially flat and mechanically stable and that the investigated compressive residual-stress cases remain within the pre-buckling regime.

For the equivalent gap coefficient *γ*, the range 0.92–0.98 is adopted. In capacitive pressure sensors, insulating layers such as silicon dioxide (*ε*_ins_ = 3.9) and silicon nitride (*ε*_ins_ = 7.9) are typically fabricated with thicknesses not exceeding 1 μm. In contrast, the gap height *g* is on the order of several micrometers. Therefore, based on Equation (22), the resulting value of *γ* is generally close to unity.

#### 3.2.1. Gap-to-Thickness Ratio, *λ*

**Figure 5:**
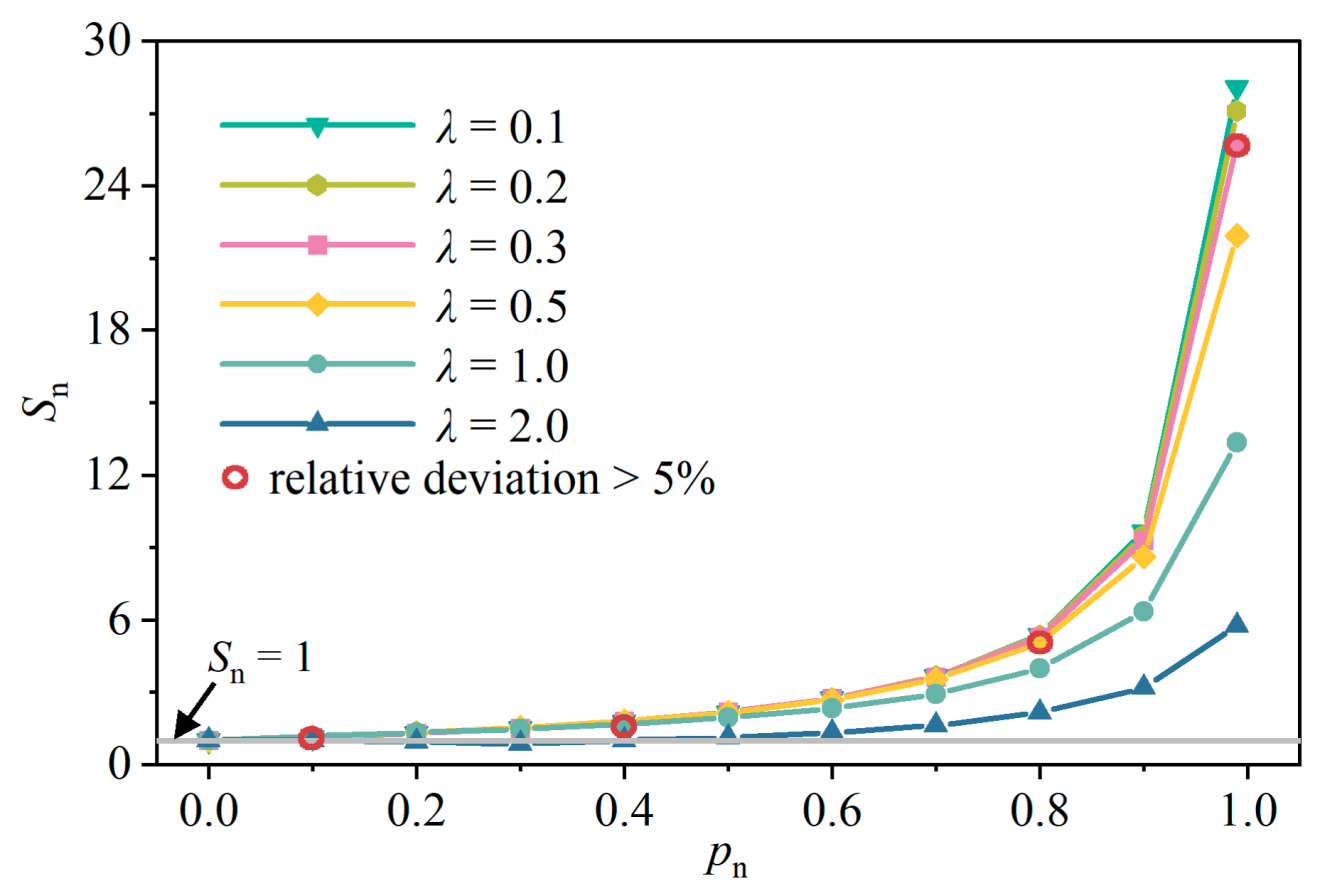
*S*_n_−*p*_n_ curves of circular-diaphragm sensors under different gap-to-thickness ratios *λ*.

A parameter sweep of the gap-to-thickness ratio *λ* is performed to examine its effect on the *S*_n_−*p*_n_ relationship. For a circular diaphragm sensor, *λ* is varied from 0.1 to 2.0, with values of 0.1, 0.2, 0.3, 0.5, 1.0, and 2.0, while *ν* = 0.28, σ^*^ = 0, and *γ* = 0.96 are held constant. The results are shown in Figure 5.

As shown in Figure 5, the *S*_n_−*p*_n_ curves corresponding to *λ* = 0.1 and 0.2 almost coincide. The maximum relative deviation between the two curves is only 3.5%, while the average relative deviation is 0.6%. This is because both cases fall within the small-deflection regime of the diaphragm, where the large-deflection governing equations reduce to the classical small-deflection equations. Consequently, *w*_0_^*^ is linearly proportional to p^*^, and the normalized deflection shape function W^*^(x^*^, y^*^) remains essentially unchanged with increasing p^*^. In this regime, the *S*_n_−*p*_n_ relationship is essentially independent of the gap-to-thickness ratio *λ*.

When *λ* > 0.2, however, the deviation of each curve from the *λ* = 0.1 reference curve gradually increases and becomes more pronounced with increasing *p*_n_. Specifically, the relative deviation exceeds 5% at *p*_n_ = 0.99 for *λ* = 0.3, at *p*_n_ = 0.8 for *λ* = 0.5, at *p*_n_ = 0.4 for *λ* = 1.0, and at *p*_n_ = 0.1 for *λ* = 2.0. Furthermore, the value of *S*_n_ for *λ* = 0.1 at *p*_n_ = 0.99 is approximately 4.9 times that of the corresponding value for *λ* = 2.0. The above phenomenon can be interpreted based on the p^*^−*w*_0_^*^ relationship described by the large-deflection governing equations. In this relationship, p^*^ can generally be approximated as a cubic-type function of *w*_0_^*^. As p^*^ increases, the increment in *w*_0_^*^ induced by a unit increment in p^*^ decreases. Therefore, under large-deflection conditions, the relative change in the reciprocal of the local capacitive gap of each diaphragm area element induced by the same Δ*p*_n_ is less pronounced than that under small-deflection conditions. This behavior is reflected in the *S*_n_−*p*_n_ relationship, where the nonlinearity of the *S*_n_−*p*_n_ curve progressively decreases as *λ* increases.

#### 3.2.2. Aspect Ratio, *AR*

**Figure 6:**
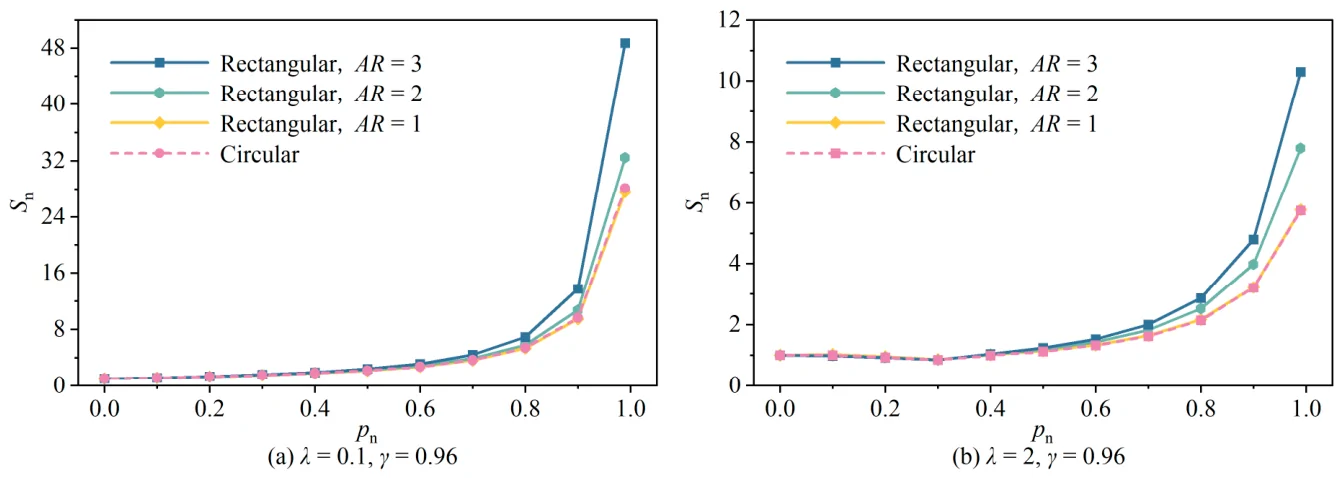
*S*_n_−*p*_n_ curves of sensors with different aspect ratios *AR*. (**a**) *λ* = 0.1. (**b**) *λ* = 2.0.

The *S*_n_−*p*_n_ curves are calculated for rectangular diaphragm sensors with aspect ratios of 1, 2, and 3, as well as for a circular diaphragm sensor, under the conditions of *γ* = 0.96, *ν* = 0.28, and σ^*^ = 0. The results obtained for *λ* = 0.1 and *λ* = 2.0 are shown in Figure 6a,b, respectively.

The results show that, under both *λ* conditions, the *S*_n_−*p*_n_ curves of the rectangular diaphragm sensor with *AR* = 1 almost completely overlap with those of the circular diaphragm sensor, with the maximum relative deviation at all data points being less than 2.0%. We consider this close agreement to result from the combined effects of the normalization procedure and the similar in-plane geometric symmetry of the two diaphragms. As *AR* further increases, the *S*_n_−*p*_n_ curves of the rectangular diaphragm sensors begin to deviate from and lie above the *AR* = 1 curve, and the deviation becomes increasingly pronounced with increasing *p*_n_. Under both *λ* conditions, the values of *S*_n_ for the *AR* = 3 curves at *p*_n_ = 0.99 are approximately 1.8 times the corresponding values for the *AR* = 1 curves. These results indicate that increasing the aspect ratio *AR* enhances the nonlinearity of the *S*_n_−*p*_n_ curve.

**Figure 7:**
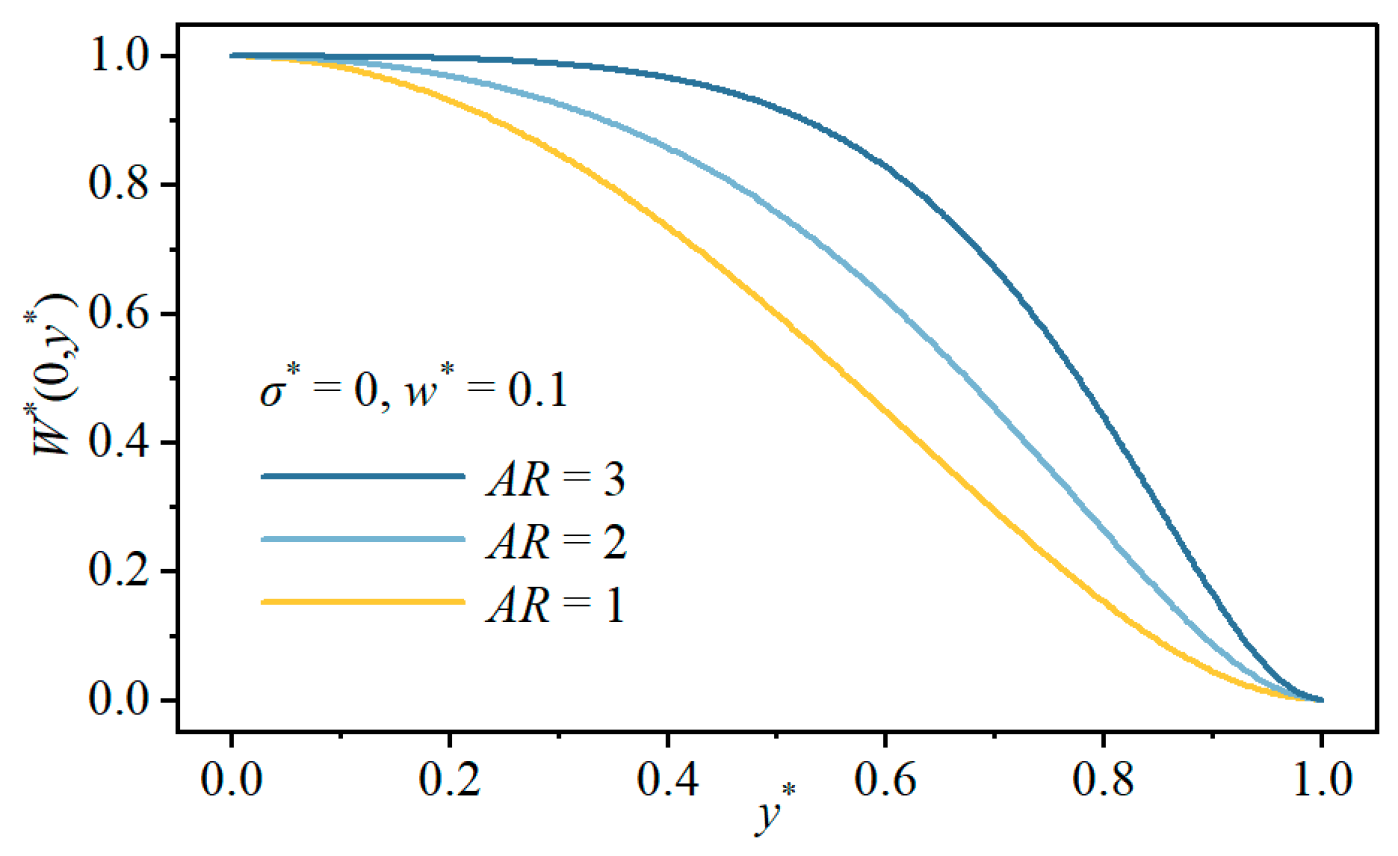
Comparison of the simulated dimensionless deflection shape functions of rectangular diaphragms with different aspect ratios *AR*.

According to Equation (32), under the small-deflection condition represented by *λ* = 0.1, the *S*_n_−*p*_n_ relationship is determined only by the deflection shape function W^*^(x^*^, y^*^), when *γ* is fixed. Figure 7 shows that the profiles of W^*^(0, y^*^) vary significantly with *AR* under *w*_0_^*^ = 0.1. As *AR* increases, a larger portion of the diaphragm exhibits deflections close to the maximum deflection, resulting in a more pronounced relative variation in the reciprocal of the local gap. This leads to a steeper *S*_n_−*p*_n_ curve for larger *AR*, thereby explaining the deviations among the curves observed in Figure 6a for the small-deflection case of *λ* = 0.1. When *λ* increases to 2, the *S*_n_−*p*_n_ curves for different *AR* values show a variation trend similar to that of the circular diaphragm, with reduced nonlinearity compared with their respective small-deflection curves.

#### 3.2.3. Poisson’s Ratio, *ν*

**Figure 8:**
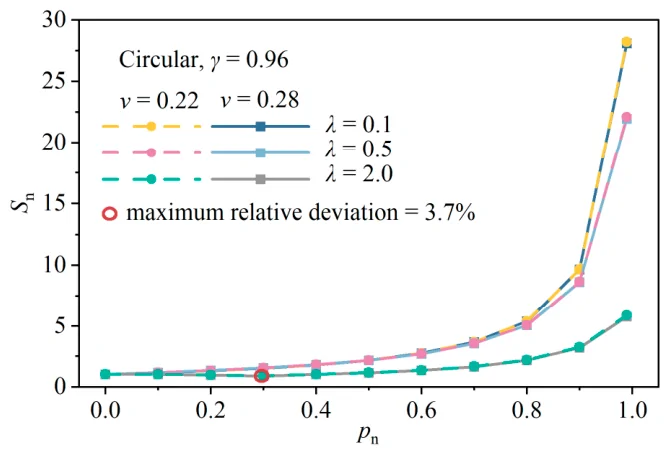
*S*_n_−*p*_n_ curves of circular-diaphragm sensors under different Poisson’s ratios *ν*.

To compare the influence of Poisson’s ratio, the *S*_n_−*p*_n_ curves of the circular diaphragm sensor are calculated for two values of *ν*, namely *ν* = 0.22 and *ν* = 0.28, while keeping σ^*^ = 0, *γ* = 0.96, and *λ* = 0.1, 0.5, and 2.0. The comparison results are shown in Figure 8.

As shown in Figure 8, the difference between the *S*_n_−*p*_n_ curves obtained under *ν* = 0.22 and *ν* = 0.28 is not significant. The maximum deviation between the curves corresponding to the two values of *ν* occurs at *p*_n_ = 0.99 for the *λ* = 2.0 case, where the deviation is only 3.7%.

This result can be interpreted based on the preceding theoretical derivation. In the dimensionless large-deflection governing equations, as well as in the relationship between p^*^ and *w*_0_^*^, Poisson’s ratio *ν* enters the relevant coefficients in the form of 1 − *ν*^2^. Although changing *ν* from 0.28 to 0.22 corresponds to a relative variation of approximately 21.4% in *ν* itself, the corresponding value of 1 − *ν*^2^ changes only from 0.9216 to 0.9516, with a relative variation of approximately 3.3%. Therefore, within this range, the influence of *ν* on both the deflection shape function W^*^(x^*^, y^*^) and the p^*^−*w*_0_^*^ relationship is relatively limited. Accordingly, Poisson’s ratio *ν* has only a minor effect on the nonlinearity of the *S*_n_−*p*_n_ curve.

#### 3.2.4. Dimensionless Residual Stress, σ^*^

**Figure 9:**
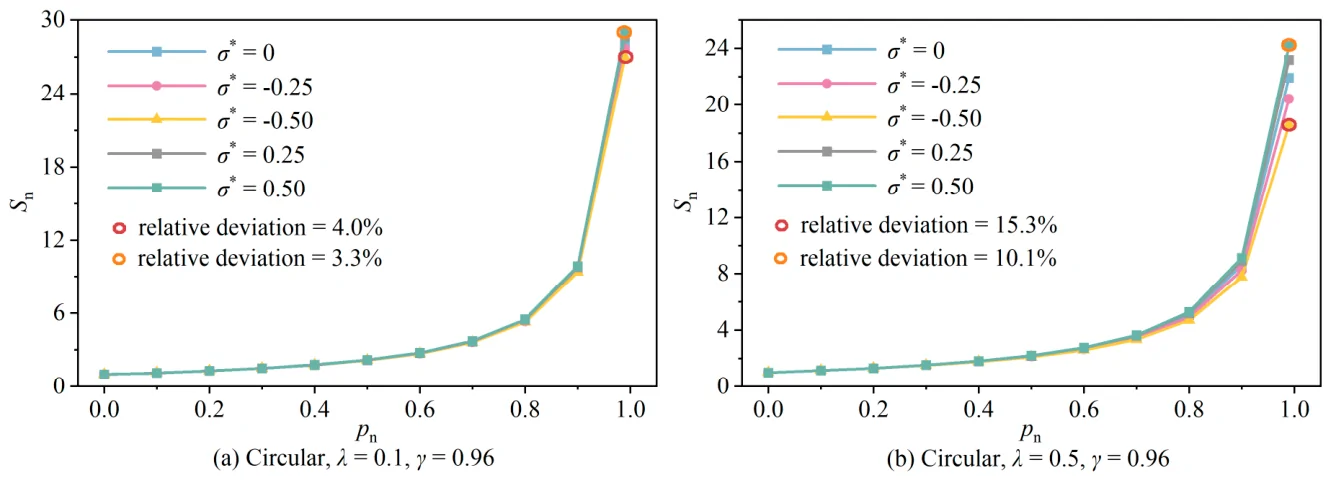
*S*_n_−*p*_n_ curves of circular-diaphragm sensors under different dimensionless residual stresses σ^*^. (**a**) *λ* = 0.1. (**b**) *λ* = 0.5.

Figure 9 compares the *S*_n_−*p*_n_ curves of circular-diaphragm sensors under different normalized residual stresses σ^*^, while *γ* = 0.96 and *ν* = 0.28 are held constant. When *λ* = 0.1, as shown in Figure 9a, the curves corresponding to σ^*^ = 0.50, 0.25, 0, −0.25, and −0.50 nearly overlap. However, the relative deviations among the curves become more pronounced as *p*_n_ approaches 1. At *p*_n_ = 0.99, the deviations of the curves for σ^*^ = −0.50 and σ^*^ = 0.50 from the curve for σ^*^ = 0 both reach their maximum values, with relative deviations of approximately 4.0% and 3.3%, respectively. When *λ* = 0.5, as shown in Figure 9b, the maximum relative deviations of the curves for σ^*^ = 0.50 and σ^*^ = −0.50 from the curve for σ^*^ = 0 both exceed 10% and occur at *p*_n_ = 0.99. Overall, compared with the case without residual stress, compressive residual stress reduces the nonlinearity of the *S*_n_−*p*_n_ curve, whereas tensile residual stress increases it. Moreover, the greater the absolute value of the residual stress, the more pronounced its influence on the nonlinear characteristics of the curve.

The reduction in the nonlinearity of the *S*_n_−*p*_n_ curve caused by compressive residual stress can be mainly attributed to its influence on the p^*^−*w*_0_^*^ relationship. Owing to the presence of compressive residual stress, for a given load increment Δ*p*_n_ near *p*_n_ = 0, the increment in *w*_0_^*^ is significantly larger than that in the stress-free case, thereby increasing the initial sensitivity *S*_0_ at *p*_n_ = 0. As *p*_n_ increases; however, the difference in the increment of *w*_0_^*^ induced by the same Δ*p*_n_ between the stressed and stress-free cases becomes relatively smaller. Consequently, after normalization by *S*_0_, the *S*_n_ values under compressive residual stress become lower than those in the stress-free case.

#### 3.2.5. Equivalent Gap Coefficient, *γ*

**Figure 10:**
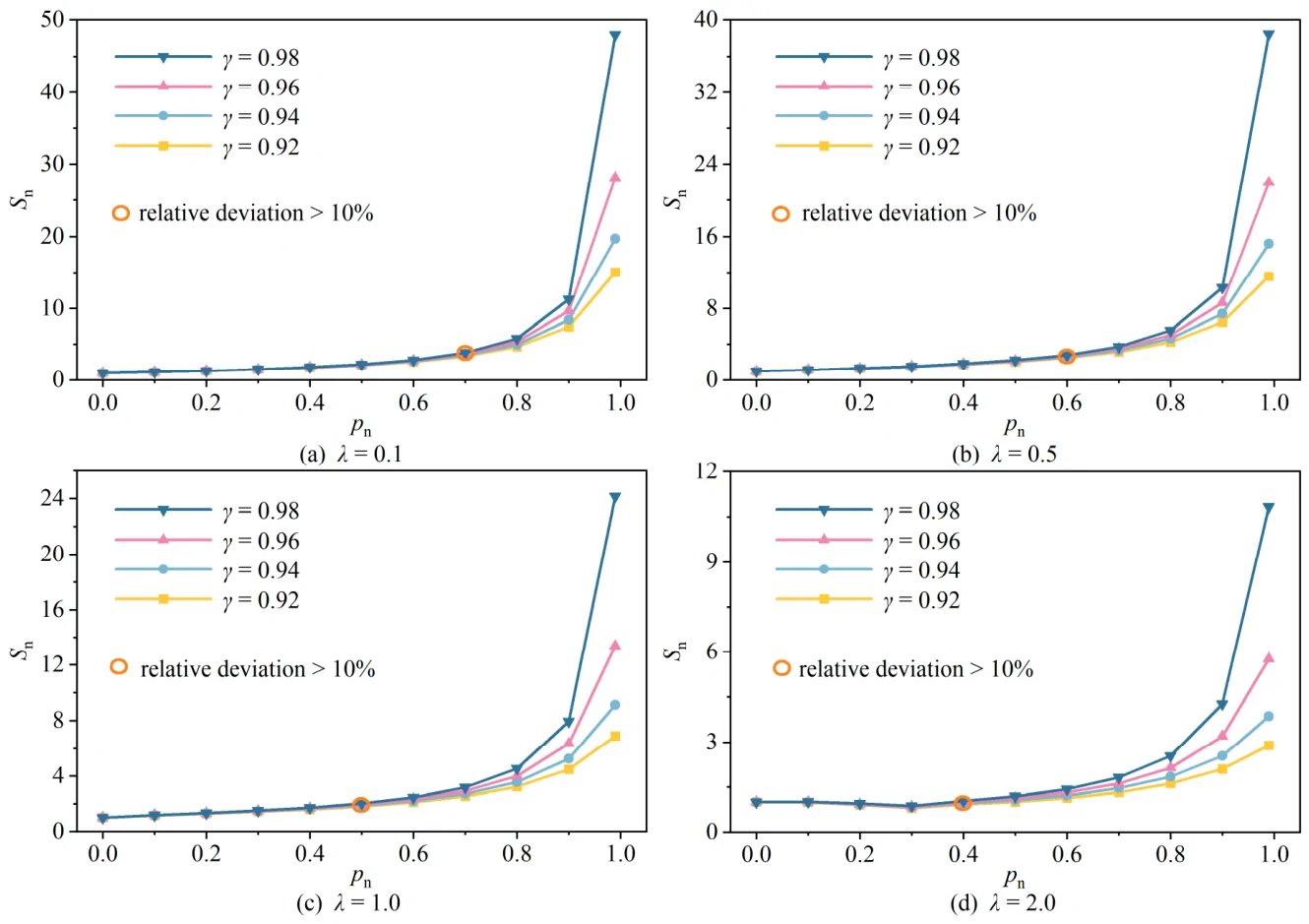
*S*_n_−*p*_n_ curves of circular-diaphragm sensors under different equivalent gap coefficients γ. (**a**) *λ* = 0.1. (**b**) *λ* = 0.5. (**c**) *λ* = 1.0. (**d**) *λ* = 2.0.

Figure 10 shows the *S*_n_−*p*_n_ curves of the circular diaphragm sensor under different values of *γ* for *λ* = 0.1, 0.5, 1.0, and 2.0, respectively. All four sets of results are obtained under the fixed conditions of *ν* = 0.28 and σ^*^ = 0. At a given *p*_n_, *S*_n_ increases with increasing *γ*, and a larger *γ* results in a more pronounced nonlinear variation of the *S*_n_−*p*_n_ curve.

Meanwhile, as *p*_n_ approaches 1, the relative deviation among the curves corresponding to different values of *γ* becomes increasingly pronounced. To further illustrate the influence of *λ*, the relative deviation of the *γ* = 0.92 curve from the *γ* = 0.98 curve is compared across Figure 10a–d. For *λ* = 0.1, the relative deviation first exceeds 10% at *p*_n_ = 0.7. As *λ* increases to 0.5, 1.0, and 2.0, the corresponding threshold shifts to *p*_n_ = 0.6, 0.5, and 0.4, respectively. This progressive shift toward lower *p*_n_ indicates that increasing *λ* amplifies the differences among the *S*_n_−*p*_n_ curves corresponding to different values of *γ*.

The increase in *S*_n_ with increasing *γ* can be explained from the perspective of a local diaphragm area element. According to Equations (21) and (26), for an arbitrary area element, the normalized capacitance increment induced by Δ*w*(*x*,*y*) under a given load increment Δ*p*_n_ is expressed by Equation (34).

(34) $$\Delta C_{n}(x,y)\propto \frac{1}{1-\gamma w(x,y)/g-\gamma \Delta w(x,y)/g}-\frac{1}{1-\gamma w(x,y)/g}$$

Based on Equations (30) and (34), the *γ*-dependent component of the local normalized sensitivity *S*_n_(*x*,*y*) is obtained.

(35) $$S_{n}(x,y)\propto \frac{1}{\left(g/\Delta w(x,y)-\gamma w(x,y)/\Delta w(x,y)-\gamma )(1-\gamma w(x,y)/g\right)}$$

Since *w*(*x*,*y*) + Δ*w*(*x*,*y*) < *g* and 0 < *γ* < 1, *S*_n_(*x*,*y*) increases with *γ* when the other variables are fixed. Consequently, the integrated normalized sensitivity *S*_n_ also increases as *γ* increases.

The increasing relative deviation among the curves for different *γ* values with increasing *λ* can also be analyzed using Equation (35). For two curves corresponding to *γ*_1_ and *γ*_2_ under the same *λ*, the ratio of the local normalized sensitivities at a given *p*_n_, *S*_n,1_(*x*,*y*)/*S*_n,2_(*x*,*y*), is given by Equation (36).

(36) $$\frac{S_{n,1}(x,y)}{S_{n,2}(x,y)}\propto \frac{1-\gamma_{2}(w(x,y)+\Delta w(x,y))/g}{1-\gamma_{1}(w(x,y)+\Delta w(x,y))/g}\frac{1-\gamma_{2}w(x,y)/g}{1-\gamma_{1}w(x,y)/g}$$

Since p^*^ can be approximately represented as a cubic function of *w*_0_^*^, increasing *λ* results in larger values of *w*(*x*,*y*)/*g* and [*w*(*x*,*y*) + Δ*w*(*x*,*y*)]/*g* at the same *p*_n_ and *p*_n_ + Δ*p*_n_. When *γ*_1_ > *γ*_2_, Equation (36) is greater than unity, and its value increases further with increasing *λ*. This explains why a larger *λ* amplifies the relative deviation between the *S*_n_−*p*_n_ curves corresponding to different values of *γ*.

Compared with the normalized sensitivity models reported by Rochus et al., Jindal et al., and Xu et al., which are mainly applicable to circular diaphragms under small-deflection conditions, the present framework provides a more general description covering both small- and large-deflection regimes. The gap-to-thickness ratio *λ* is explicitly incorporated to characterize the diaphragm-deflection regime. At small values of *λ*, such as *λ* = 0.1 and 0.2, the *S*_n_−*p*_n_ curves approach the results obtained under the small-deflection approximation. When *λ* > 0.3, however, large-deflection effects become increasingly pronounced, resulting in marked deviations from the small-deflection results. The framework also incorporates the effects of diaphragm aspect ratio *AR*, Poisson’s ratio *ν*, and normalized residual stress σ^*^. Although the equivalent gap coefficient *γ* was previously introduced by Xu et al., its influence is further examined here across different deflection regimes represented by *λ* = 0.1, 0.5, 1.0, and 2.0. This analysis reveals how the effect of *γ* varies as the sensor response transitions from small- to large-deflection conditions.

For practical MEMS capacitive pressure sensor design, the proposed dimensionless framework enables the nonlinearity trends of sensors with different structural and material parameter combinations to be rapidly assessed and compared through their governing dimensionless parameters, thereby facilitating preliminary screening without requiring a complete point-by-point calculation of the *S*−*p*_diff_ relationship for every candidate design. Furthermore, a family of normalized *S*_n_−*p*_n_ characteristic curves can be established from a limited number of representative cases. For a sensor sharing the same dimensionless parameters as an established curve, once its contact pressure *p*_c_ and local sensitivity *S*_0_ near the initial operating point are determined—both of which are also required in conventional sensor analysis—the sensitivities at the remaining operating points can be obtained from *S* = *S*_0_*S*_n_(*p*_n_) through curve lookup and multiplication, rather than by repeatedly calculating *C*(*p*_diff_) and *C*(*p*_diff_ + Δ*p*) at each pressure point. In addition, the degree of sensitivity nonlinearity can be directionally adjusted by modifying the corresponding dimensional structural and material parameters according to the identified effects of the governing dimensionless parameters on the *S*_n_−*p*_n_ curves. These findings improve the efficiency of candidate-design screening and sensitivity evaluation, reduce repeated point-by-point capacitance and sensitivity calculations, and provide directional guidance for iterative optimization.

## 4. Conclusions

In this study, the nonlinear sensitivity characteristics of capacitive pressure sensors operating in the non-contact mode are systematically investigated based on the large-deflection governing equations. A normalized analytical model for the output of capacitive pressure sensors is established. It is demonstrated that, when the dimensionless parameters *λ*, *AR*, *ν*, σ^*^, and *γ* are identical, capacitive pressure sensors with different combinations of structural and material parameters exhibit similar *S*_n_−*p*_n_ curves. Using the indirect computation method, it is further verified that the average relative deviation among the *S*_n_−*p*_n_ curves of sensors with different dimensional parameters but identical dimensionless parameters does not exceed 0.05%. The effects of the dimensionless parameters on the nonlinear characteristics of normalized sensitivity are also revealed. Among these parameters, increasing *γ* or *AR* tends to increase the degree of nonlinearity of the *S*_n_−*p*_n_ curve, whereas increasing *λ* tends to reduce it. Within the investigated pre-buckling range, compressive residual stress reduces the degree of nonlinearity, whereas tensile residual stress increases it; in both cases, the influence becomes more pronounced as the magnitude of the dimensionless residual stress increases. The influence of Poisson’s ratio *ν* is relatively limited.

Overall, the proposed dimensionless framework provides a systematic and unified approach for comparing, predicting, and tailoring the nonlinear sensitivity characteristics of non-contact capacitive pressure sensors, thereby facilitating sensor design evaluation and optimization.

## Figures and Tables

**Table 1:** Structural and material parameters of sensors for verifying the similarity of the *S*_n_−*p*_n_ curves.

No.	*h* (μm)	*a* (μm)	*b* (μm)	*g* (μm)	*t* (μm)	*ν*	*E* (GPa)	*σ*_0_ (MPa)	*ε*_ins_
1	3	*R* = 300	*R* = 300	2.1	0.30	0.28	170	−3.40	3.9
2	6	*R* = 500	*R* = 500	4.2	0.60	0.28	170	−4.90	3.9
3	1	200	400	1.8	0.39	0.28	170	−2.13	3.9
4	2	300	600	3.6	0.78	0.28	170	−3.78	3.9

## Data Availability

The original contributions presented in this study are included in the article. Further inquiries can be directed to the corresponding author.
